# Multi-Faceted Roles of ERCC1-XPF Nuclease in Processing Non-B DNA Structures

**DOI:** 10.3390/dna2040017

**Published:** 2022-10-11

**Authors:** Tonia T. Li, Karen M. Vasquez

**Affiliations:** Division of Pharmacology and Toxicology, Dell Pediatric Research Institute, College of Pharmacy, The University of Texas at Austin, 1400 Barbara Jordan Boulevard, Austin, TX 78723, USA

**Keywords:** non-B DNA structure, DNA repair, ERCC1-XPF, genetic instability

## Abstract

Genetic instability can result from increases in DNA damage and/or alterations in DNA repair proteins and can contribute to disease development. Both exogenous and endogenous sources of DNA damage and/or alterations in DNA structure (e.g., non-B DNA) can impact genome stability. Multiple repair mechanisms exist to counteract DNA damage. One key DNA repair protein complex is ERCC1-XPF, a structure-specific endonuclease that participates in a variety of DNA repair processes. ERCC1-XPF is involved in nucleotide excision repair (NER), repair of DNA interstrand crosslinks (ICLs), and DNA double-strand break (DSB) repair via homologous recombination. In addition, ERCC1-XPF contributes to the processing of various alternative (i.e., non-B) DNA structures. This review will focus on the processing of alternative DNA structures by ERCC1-XPF.

## 1. Introduction

DNA damage can occur from both endogenous and exogenous sources, such as oxidative damage and UV light, respectively [1,2]. DNA damage that is left unrepaired, or is repaired in an error-generating fashion, can lead to genetic instability which can contribute to the initiation and/or progression of various diseases, such as cancer [3,4]. Organisms possess DNA repair proteins that are vital in the maintenance of the genome. ERCC1-XPF is one such protein complex.

ERCC1-XPF is an endonuclease well known for its role in the nucleotide excision repair (NER) mechanism. In this repair pathway, ERCC1-XPF participates in the removal of bulky DNA adducts, such as 6–4 photoproducts caused by UV radiation [5–7]. Upon recognition of the damage and following the unwinding of the DNA duplex around the site of damage, ERCC1-XPF cleaves on the 5′ side of the damaged strand. Another NER nuclease, XPG, cleaves on the 3′ side of the lesion on the same strand [5–8]. Depending on the type of damage, cleavage by ERCC1-XPF and XPG typically results in the removal of a 25–30 nucleotide fragment containing the damage [8]. In addition to its role in NER, ERCC1-XPF is also known to participate in DNA double-strand break (DSB) repair, DNA interstrand-crosslink (ICL) repair, base excision repair (BER), and telomere maintenance [5–8].

In humans, individuals with deficiencies in XPF can develop disorders such as Xeroderma Pigmentosum (XP), XFE progeroid syndrome, Fanconi anemia (FA), and Cockayne syndrome (CS) [5–8]. Individuals with deficiencies in ERCC1 have been reported to develop cerebro-oculo-facio-skeletal syndrome [5–7]. Mice with deficiencies in ERCC1 show signs of accelerated aging, while deletion of ERCC1 in mice is lethal [5–7]. Several studies have also shown that ERCC1-XPF can act outside of canonical DNA repair pathways. For example, ERCC1-XPF is involved in the mutagenic processing of alternative non-B DNA structures. Herein, we focus on the roles of ERCC1-XPF in the processing of several non-B DNA structures.

## 2. Non-B DNA

**Figure 1.**
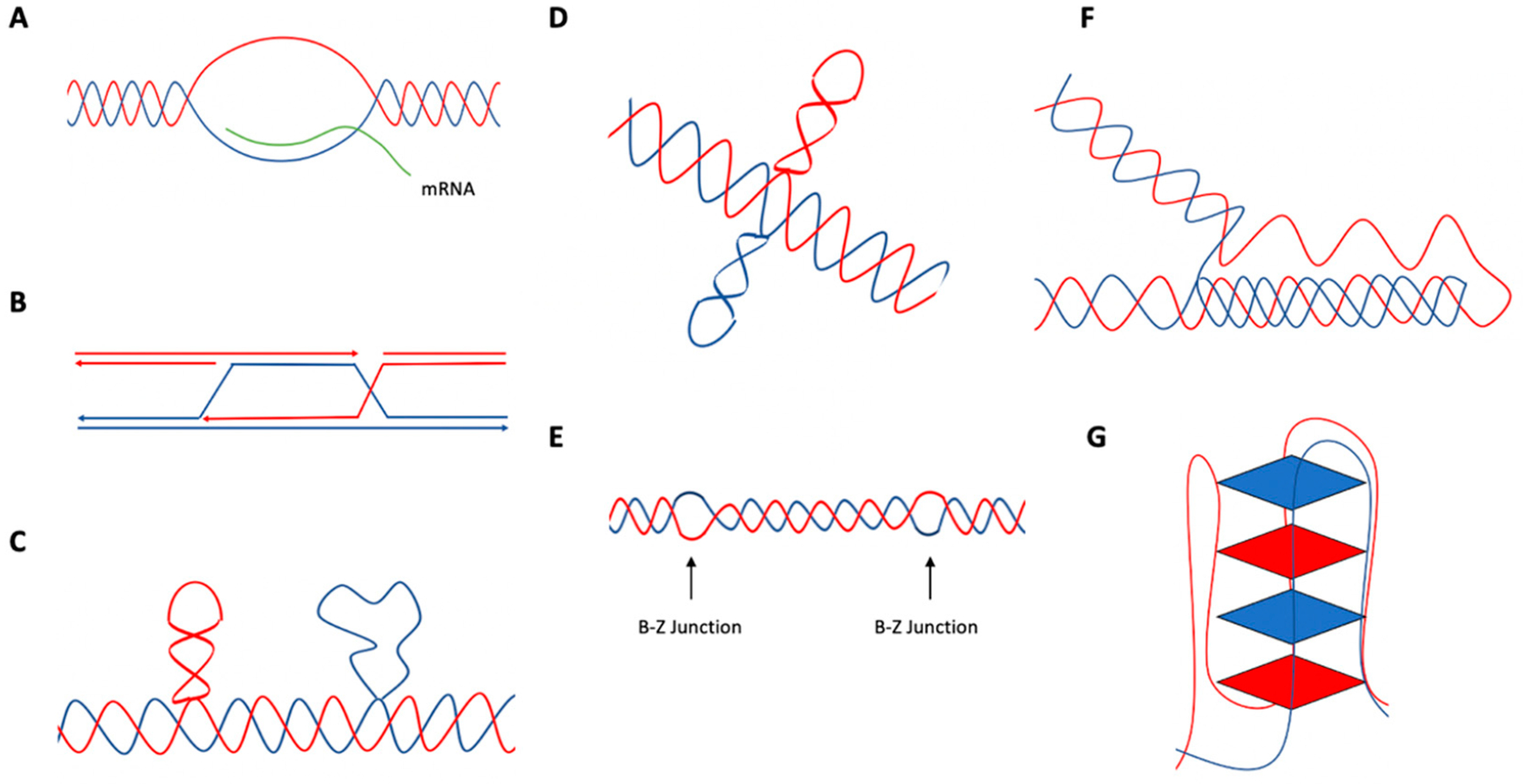
Schematic of non-B DNA structures. (**A**) R-loop, (**B**) D-loop, (**C**) hairpin/stem-loop and slipped DNA (formed at inverted repeat sequences), (**D**) cruciform DNA formed at inverted repeat sequences, (**E**) Z-DNA (formed at alternating purine-pyrimidine sequences), (**F**) H-DNA (formed at polypurine and polypyrimidine DNA sequences with mirror symmetry), (**G**) G-quadruplex (G4) DNA (formed at G_≥3_N_x_G_≥3_N_x_G_≥3_N_x_G_≥3_ DNA sequences). Adapted from Wang and Vasquez (2014) [12].

Repetitive sequences in genomic DNA are abundant (>50% of the human genome), and these sequences have the potential to adopt alternative (non-B DNA) structures that differ from the canonical right-handed B-form DNA. Numerous alternative structures have been characterized, including, but not limited to, R-loops, D-loops, hairpin/stem loops, slipped DNA, cruciform DNA, Z-DNA, H-DNA, and G-quadruplex (G4) DNA (Figure 1) [9–12]. Non-B DNA structures can form during processes that involve unwinding of the DNA helix, such as replication, transcription, and DNA repair. This affords single-stranded DNA the opportunity for intrastrand base pairing, forming alternative structures such as hairpins [9,11,12]. These cellular processes also generate negative supercoiling, which provides energy for the formation and stabilization of alternative DNA structures [9,11,12]. Once formed, these structures can contribute to a variety of biological processes, such as transcriptional regulation, chromatin remodeling, and genetic instability, thereby contributing to disease etiology and evolution [11–13].

Importantly, non-B DNA-forming sequences have been found to co-localize with translocation breakpoint “hotspots” in human cancer genomes, and are also enriched at sites of DSBs and deletions [9,11,12,14,15], implicating them in disease etiology. For example, the human *BCL-2* and *c-MYC* genes contain DNA sequences with the capacity to adopt non-B DNA structures that overlap with areas prone to breakage and chromosomal translocations found in lymphomas and leukemias [9,11,12]. These mutagenic structures are resolved by various DNA repair proteins that may act within or outside of their canonical repair pathways. One such repair protein complex, ERCC1-XPF, will be discussed below with a focus on its roles in processing non-B DNA structures.

## 3. R-Loops

R-loops are comprised of DNA-RNA hybrids that can form when RNA transcripts bind to complementary DNA templates, resulting in a three-stranded nucleic acid structure with a single-stranded (ssDNA) section [16–19] (Figure 1A). R-loops can form as intermediates in various cellular processes, such as transcription, immunoglobulin class switching, and replication [16–19].

R-loops have been found to increase genomic instability by a variety of mechanisms [16–21]. For example, R-loop formation creates a ssDNA region that may be more prone to DNA-damaging agents and/or cleavage than dsDNA [17–20]. Proteins that recognize the R-loop structure, such as activation-induced cytidine deaminase (AID), may also contribute to genetic instability [16–21]. Additionally, the formation of R-loops during transcription may interfere with DNA replication, leading to collisions between R-loops and progressing replication forks [16–21]. R-loops have been implicated in the development of various neurodegenerative diseases, including, but not limited to, Friedreich ataxia and Fragile X syndrome [16,20,22]. R-Loops have been found to form on expanded repeats in the *FMR1* and *FMX* genes, known to be associated with Friedreich ataxia and Fragile X syndrome [16,20,22]. R-loops within the *FMR1* and *FXN* genes have been found to colocalize with H3K9me2, a repressive chromatin mark, and have been shown to impede RNA polymerase II, thereby inhibiting gene transcription [16,22].

Several proteins have been implicated in the resolution of R-loops. For example, RNase H can remove the RNA in a DNA-RNA complex [20,23–26]. Senataxin (SETX) and its yeast homolog Sen1 can unwind RNA-DNA complexes [20,27,28]. Topoisomerase I may suppress R-loop formation by relaxing DNA supercoiling [20,29] and promoting the assembly of mRNPs (mRNA-particle complexes) in an ASF/SF2 (pre-mRNA splicing factor)-dependent fashion [29]. Additionally, the NER nucleases ERCC1-XPF and XPG have been found to cleave R-loops, leading to the formation of DSBs [30,31].

**Figure 2.**
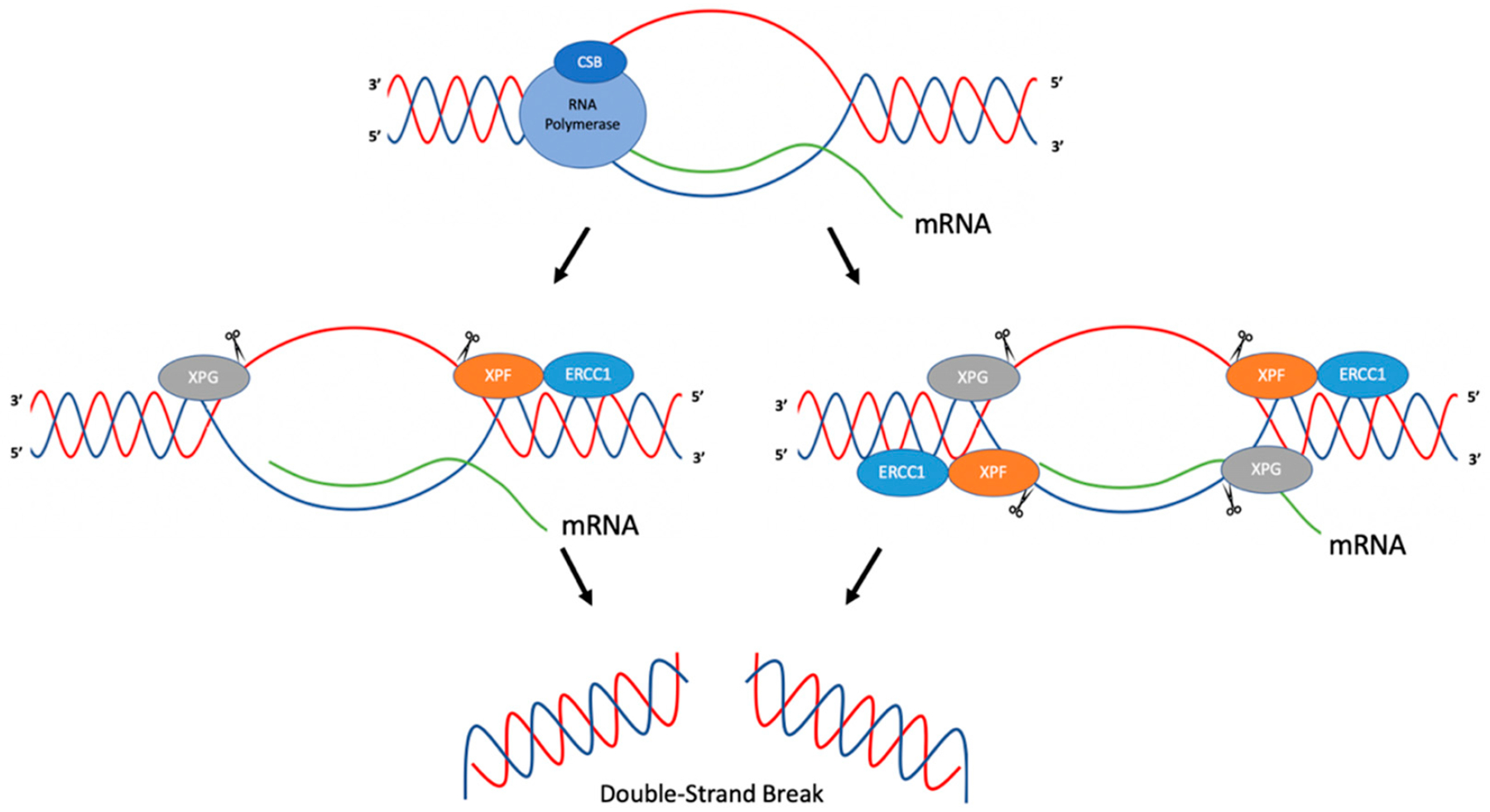
Proposed mechanism for the cleavage of R-loops. ERCC1-XPF and XPG are recruited to R-loops and can cleave the junctions between the R-loop and the duplex DNA. ERCC1-XPF can cleave both the template and non-template strand, ultimately resulting in DSBs. Adapted from Sollier et al. (2014) [31] and Tian and Alt (2000) [30].

Tian and Alt (2000) have demonstrated that ERCC1-XPF and XPG can cleave R-loops in immunoglobulin switch regions during class switch recombination [30]. In this study, plasmid constructs were created where transcription of immunoglobulin switch regions was inducible by T7 RNA polymerase. R-loop formation was determined via P1 nuclease cleavage in vitro after transcription was induced. In order to determine the activity of ERCC1-XPF and XPG on R-loops, an R-loop substrate was created using oligonucleotides and an in vitro transcribed RNA strand, and incubated with purified human recombinant ERCC1-XPF and XPG proteins [30]. In this system, ERCC1-XPF was found to cleave at the 5′ side of the R-loop duplex junction in both template and non-template strands. XPG was found to cleave at the 3′ side of the R-loop duplex junction on only the non-template strand. Studies were then performed in order to determine whether ERCC1-XPF and XPG could process R-loops formed in transcribed immunoglobulin switch regions. Using plasmid constructs, S regions were transcribed and incubated with ERCC1-XPF and XPG. Cleavage was observed to be more efficient on the non-template strand than on the template strand, though both were observed. Two mechanisms of R-loop processing in immunoglobulin switch regions were proposed. In the first, ERCC1-XPF and XPG cleave both template and non-template strands of the R-loop, creating a DSB, which could then result in class switch recombination or deletions in the switch region upon repair. In the second model, ERCC1-XPF and XPG cleave on the non-template strand, creating single-strand breaks. These breaks could then lead to DSBs during replication by replication fork collapse, which, during repair, could result in class switch recombination products [30] (Figure 2).

In another study, Sollier et al. (2014) found that R-Loops, induced by the absence of RNA processing factors such as Aquarius (AQR, an RNA/DNA helicase), could be resolved by ERCC1-XPF and XPG. Cells depleted of AQR showed a twofold increase in RNA-DNA hybrids compared to wild-type cells. This was ascertained through the use of S9.6, a monoclonal antibody used to detect RNA-DNA hybrids. This increase in RNA-DNA hybrids, suggested to be R-loops, led to increased DNA damage responses (DDR) as assessed by upregulation of γH2AX; phosphorylation of KAP1, CHK1, and RPA-2; and increased DSB formation as measured by neutral comet assays. The authors speculated that nucleases that cleave flap structures, such as XPG and ERCC1-XPF, may process R-loops due to the structural similarities of the substrates [31]. Using XPG- and XPF-deficient fibroblasts from XP patients and isogenic cell lines with added wild-type XPG and XPF, knockdown of AQR in cell lines supplemented with wild-type XPF or XPG resulted in phosphorylation of KAP1, while phosphorylation of KAP1 was reduced in the XPF- and XPG-deficient cell lines, which suggested roles for XPF and XPG in DSB formation and the processing of R-loops [31]. This was further confirmed through an immunofluorescence assay using nuclease inactive forms of XPG or XPF, where upon knockdown of AQR, the levels of γH2AX were reduced relative to the levels in wild-type cells [31]. In order to determine whether similar effects were observed in the absence of other RNA processing factors, XPG was knocked down in cells depleted of ASF (a splicing factor), SETX (an RNA/DNA helicase), or cells treated with camptothecin (CPT; a topoisomerase I inhibitor). Similar results were observed where knockdown of XPG reduced DNA damage responses. Based on these findings, the authors suggested that ERCC1-XPF and XPG were involved in the processing of R-loops [31]. Because ERCC1-XPF and XPG are nucleases that function in NER, additional proteins in the NER pathway were studied in order to determine whether they also had roles in processing R-loops. Depletion of XPA, XPB, or XPD reduced the DDR in the absence of AQR, implicating NER factors in the processing of R-loops. Further experiments were conducted in order to determine whether global genome repair (GG-NER) and/or transcription coupled repair (TC-NER) were involved in processing R-loops. Depletion of XPC (GG-NER) and CSB (TC-NER) were studied in conjunction with depletion of AQR. Phosphorylation of KAP1 was reduced when cells were depleted of CSB, but not XPC, suggesting that proteins in TC-NER process R-loops, leading to the formation of DSBs [31]. One proposed mechanism suggests that upon RNA polymerase stalling, CSB recruits XPF and XPG to process R-loops [31] (Figure 2). A limitation to such studies is that direct evidence for the formation of R-loops in cells is technically challenging to obtain, and thus often only indirect evidence is provided.

## 4. D-Loops

Displacement loops (D-loops; Figure 1B) are three-stranded DNA structures which, among other things, can serve as intermediates during homologous recombination. Homologous recombination, a highly conserved mechanism for DNA repair, uses a homologous chromosome or sister chromatid for the repair of DSBs. Homologous recombination is initiated with Replication Protein A (RPA) binding to ssDNA. Rad51 displaces RPA and forms a filament on the ssDNA molecule in order to search for homology within a homologous chromosome or a sister chromatid, at which time strand invasion occurs [32,33]. This creates a structure that contains a new heteroduplex from the invading ssDNA binding to a complementary sequence, as well as a displaced ssDNA from the DNA duplex (i.e., a D-loop) [32,33]. Various mechanisms have been identified where ERCC1-XPF participates in the resolution of D-loops, such as DSB repair and synthesis-dependent strand annealing (SDSA) [34,35].

**Figure 3.**
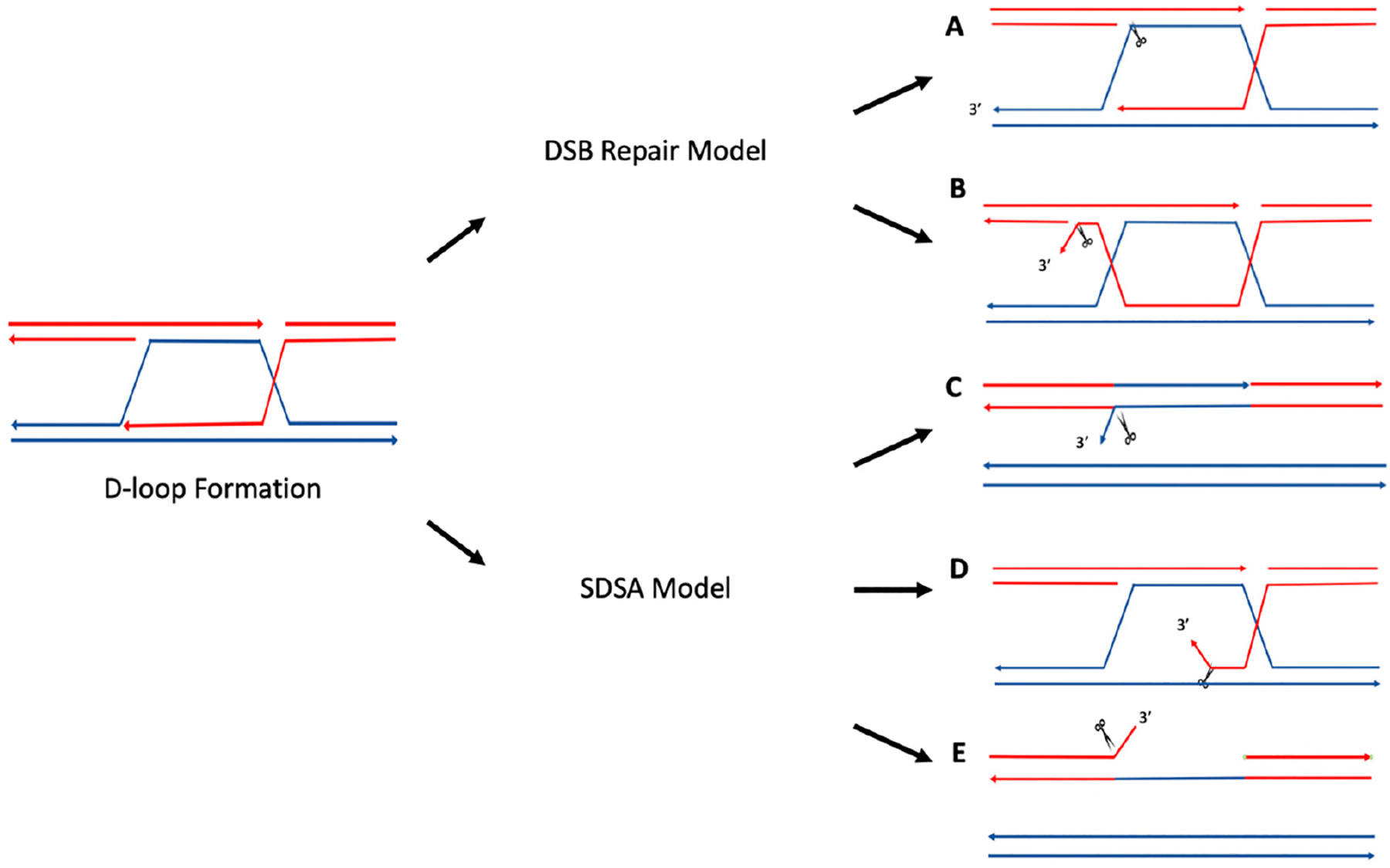
Cleavage of D-loops by Rad10-Rad1. (**A**) Rad10-Rad1 cleaves the D-loop to create a nicked Holliday junction or a single Holliday junction. (**B**) Rad10-Rad1 cleaves 3′ overhangs creating a double Holliday junction. (**C**) Removal of the 3′ flap of newly synthesized DNA. DNA synthesized past the point of homology is removed by Rad10-Rad1. (**D**) Removal of non-homologous 3′ tails to initiate DNA synthesis. (**E**) Removal of non-homologous 3′ tail on the non-invading strand. Cleavage by Rad10-Rad1 is represented by scissors. Adapted from Giaccherini and Gaillard (2021) [34], Mazón et al. (2012) [35], and Lyndaker and Alani (2009) [38].

In the DSB repair model, Holliday junctions form during strand invasion by the 3′ ssDNA, creating a D-loop intermediate; this 3′ ssDNA can then anneal to the other end of the double-strand break, creating a double Holliday junction after polymerization. Resolution of these junctions may result in crossover events, which have been shown to cause genetic instability by generating deletions, translocations, etc. [35,36]. While several protein complexes are able to resolve these Holliday junctions (e.g., Sgs1-Top3-Rmi1 in yeast and BLM-TOPIIIα-RMI1-RMI2 in humans for non-crossover events [35–37], and Mus81-Mms4 and Yen1 in yeast and MUS81-EME1, GEN1, and SLX1-SLX4 in humans for potential crossover events [35]), it has been proposed that the yeast complex Rad10-Rad1 (ERCC1-XPF in mammals) can cleave D-loops to create intermediate products that can be resolved by Mus81-Mms4 or Yen1 in yeast [35]. In a homology-dependent repair assay in yeast, the formation of ectopic joint molecules was examined in the presence of a *mus81Δyen1Δ* double mutant and a *mus81Δrad1Δyen1Δ* triple mutant. Results showed higher accumulation of ectopic joint molecules in the *mus81Δyen1Δ* double mutant than in the *mus81Δrad1Δyen1Δ* triple mutant, suggesting that Rad10-Rad1 cleaved D-loops at the heterology barrier. As depicted in Figure 3A, cleavage of the D-loop by Rad10-Rad1, followed by DNA synthesis and ligation, could create an intermediate product with a single/nicked Holliday junction resolved by Mus81-Mms4 and/or Yen1 [35]. It was speculated that Rad10-Rad1 may also cleave 3′ flaps created when DNA is synthesized beyond the point of homology during polymerization, prior to annealing (Figure 3B) [35].

ERCC1-XPF (Rad10-Rad1 in yeast) has been found to cleave single-stranded flap structures at heterology barriers. During SDSA, DNA synthesis occurs from the invading ssDNA, and after DNA synthesis is completed, the invading strand can separate from the D-loop and anneal to the other end of the break. This results in DNA repair without crossover events [34–36,39,40]. As depicted in Figure 3C–E, if 3′ nonhomologous tails are generated in this process, they can be removed by ERCC1-XPF [34,38]. For example, it has been shown that Rad1, the yeast homolog of XPF, can remove nonhomologous DNA ends during recombination. Rad1 mutants were unable to complete recombination when there was a nonhomologous section of 60 base pairs, yet were able to complete the reaction once homology was restored [41].

Studies have demonstrated that the recruitment of Rad10-Rad1 to 3′ nonhomologous tails can be mediated by the mismatch repair protein complex, Msh2-Msh3 [38,42–44]. In *Saccharomyces cerevisiae*, recombination substrates were used to determine the roles of Msh2 and Msh3 in homologous recombination. Various centromeric plasmids, containing a HO restriction site, were used to measure DSB repair. Upon cleavage at the HO cut site, plasmids possessed either ends with near perfect homology to the donor DNA or ends with nonhomology composed of varying lengths. With HO-induced gene conversion, plasmids with homology did not require Rad1, Msh2, or Msh3 for successful DSB repair. In the absence of Rad1, Msh2, Msh3, Msh2 and Rad1, or Msh3 and Rad1, plasmids containing nonhomology showed a significant reduction in DSB repair relative to the control. These results implicate Msh2 and Msh3 in the removal of nonhomologous tails, in conjunction with Rad10-Rad1 [44]. Subsequent studies have determined that Rad1, Msh2, and Msh3 can remove nonhomologous tails of 30 bp or more (e.g., up to 610 nucleotides in this study) [42].

## 5. Hairpin/Stem-Loops and Cruciform DNA

Hairpin/stem loop or cruciform DNA structures (Figure 1C,D) can form at inverted repeat sequences where intrastrand hydrogen bonding can occur [9,11,45]. Depending on the inverted repeat sequence, these structures may or may not contain a loop of unpaired nucleotides. The formation of hairpin/cruciform structures involves processes in which DNA exists in a single-stranded state (e.g., during replication, transcription, or DNA repair) [9,45,46]. If left unrepaired or repaired in an error-generating fashion, hairpin and cruciform structures can lead to genetic instability. For example, during replication, the ssDNA forming a hairpin may lead to a deletion or expansion within the newly synthesized DNA, depending on whether it is in the template or nascent strand [9,46]. Inverted repeat sequences greater than 8 bp are abundant, with an estimated occurrence of ~1 in 5,600 bp in the human genome, whereas ideal cruciform-forming sequences are found ~1 in 41,800 bp in the human genome [47]. These sequences have also been shown to be enriched at mutation hotspots in cancer genomes, implicating them in cancer etiology [14].

Hairpin/cruciform-forming AT-rich palindromic sequences in chromosomes 11q23 and 22q11 can induce genetic instability in the form of duplications, deletions, and translocations in the human genome [9,48,49]. Deletions within chromosome 22q11 can result in DiGeorge syndrome, velocardiofacial syndrome, and conotruncal anomaly face syndrome [49]. Duplications within chromosome 22q11 can cause Cat Eye syndrome [49]. The t (11;22) (q23;q11) translocation is a recurrent translocation found within the human genome. Balanced carriers of this translocation have no symptoms; however, their offspring typically experience fertility issues and chromosomal imbalances. In severe cases, offspring will develop Emmanuel syndrome, which manifests in developmental disorders and physical anomalies [48,49].

**Figure 4.**
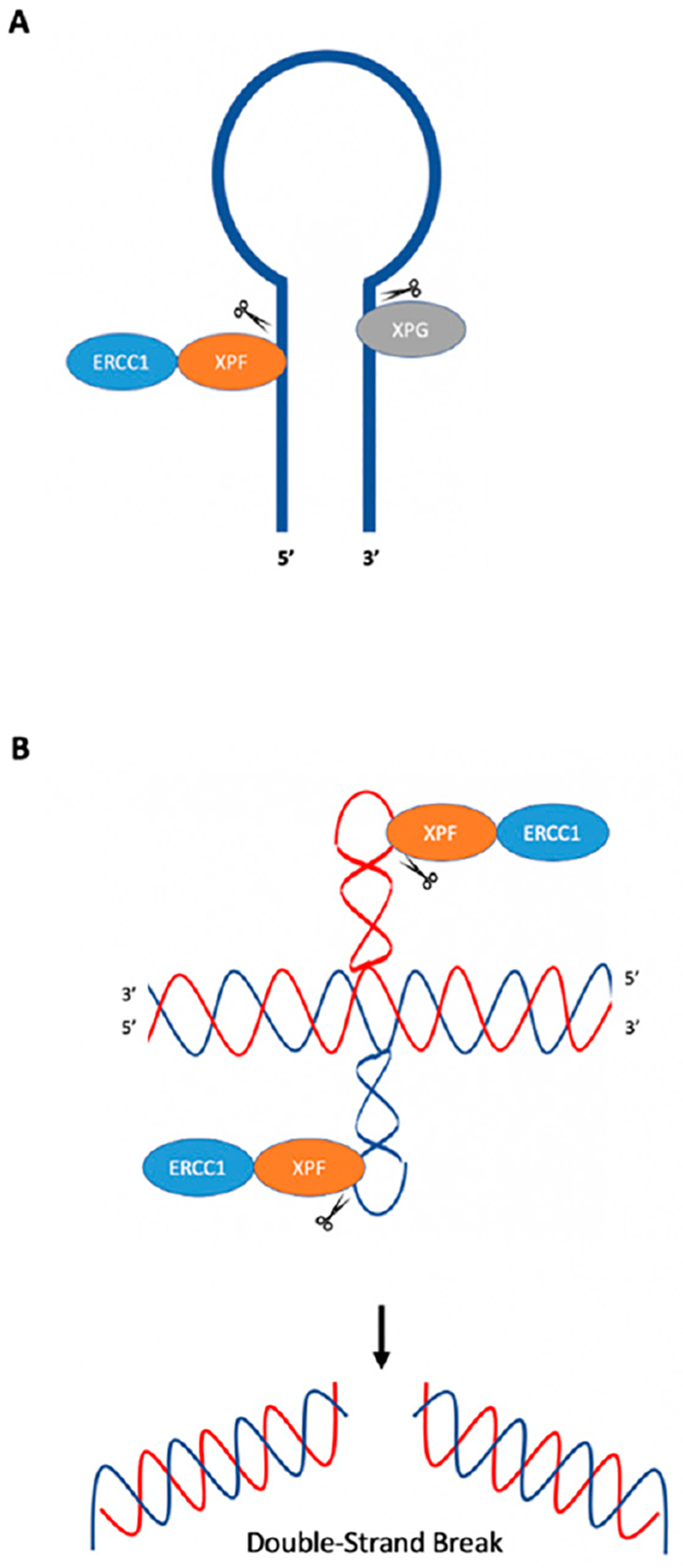
Cleavage of hairpin and cruciform structures by ERCC1-XPF. (**A**) ERCC1-XPF cleaves the stem at the 5′ side of hairpins, while XPG cleaves the 3′ side. (**B**) ERCC1-XPF cleaves cruciform loops. Cleavage of both loops may result in DSBs that can be processed in an error-generating fashion, resulting in genetic instability. Adapted from de Laat et al. (1998) [51], Sijbers et al. (1996) [52], and Lu et al. (2015) [53].

In *E. coli*, the SbcCD nuclease can cleave hairpins near the loop and stem junction at the 5′ end of the loop [9], which can result in DSBs that can be repaired through recombination [46,50]. Rad50 and Mre11 (yeast and mammalian homologs to SbcC and SbcD) act similarly on hairpin structures [9]. Studies have demonstrated ERCC1-XPF endonuclease activity on oligonucleotides which form stem–loop structures using purified ERCC1-XPF from Chinese hamster ovary (CHO) cells or human recombinant purified ERCC1-XPF. ERCC1-XPF was shown to cleave the stem of the structure on the 5′ side two, three, and four phosphodiester bonds away from the loop [51,52], while XPG cleaved the 3′ side of the loop near the intersection between the stem and loop [52]; see Figure 4A.

Lu et al. (2015) have demonstrated that short inverted repeats, which can adopt cruciform structures, induce genetic instability in the form of large deletions and insertions in both replication-dependent and replication-independent mechanisms [53]. In replication-dependent mutagenesis, Lu et al. found that cruciform structures caused fork stalling during replication, which could lead to DSBs [53]. Interestingly, in replication-independent mutagenesis, ERCC1-XPF was recruited to cruciform structures and cleaved on the 3′ side of the cruciform loop, rather than 5′ of the single-strand double-strand junction, as might be expected from its cleavage patterns on canonical stem–loop or ”bubble” substrates. This difference in the cleavage pattern may be due to the sequences/structure of the cruciform. Genetic studies have confirmed a role for Rad1 (XPF) in the mutagenic processing of cruciform DNA in both yeast and human cells. When a cruciform-forming inverted repeat sequence was inserted downstream of a *URA3* gene mutation reporter in yeast, mutations were substantially reduced in the absence of Rad1. These findings were verified in human cell extracts using purified human recombinant protein, where it was found that ERCC1-XPF cleaved cruciform structures in vitro several nucleotides from the loop on the 3′ side (Figure 4B). DSBs could be generated by cleavage of both cruciform loops at the 3′ side by ERCC1-XPF, which could then be resolved through microhomology-mediated end-joining, resulting in large deletions [53]. Habraken et al. (1994) demonstrated that Rad1, the yeast homolog of human XPF, cleaved four-way junctions (Holliday junction) in an in vitro assay using four hybridized oligonucleotides (termed X12); however, the results have been debated [54,55]. In another study, Bardwell et al. (1994) demonstrated Rad10-Rad1 activity at junctions between duplex and ssDNA [56]. West (1995) advised caution when interpreting results using the X12 construct, as it may contain single-stranded regions, and proposed that Holliday junctions themselves are not the targets of Rad1, but instead that the targets are the junctions between duplex and ssDNA, referencing the results from Bardwell et al. (1994) [55,56].

## 6. Z-DNA

As depicted in Figure 1E, Z-DNA is a left-handed structure where segments of DNA containing alternating purine-pyrimidine sequences adopt a *syn-* and *anti*-configuration, respectively, resulting in a “zigzag” conformation [9,11,57]. There are two sections of ssDNA on either side of the Z-DNA structure comprising the B-Z junction, where it has been reported to have increased sensitivity to S1 nuclease [57–59]. Repetitive CG sequences are preferred Z-DNA-forming sequences; however, these sequences are not as abundant as other dinucleotides in eukaryotic genomes. Instead, sequences of CA/TG repeats are found widely in eukaryotic genomes, and are located in a variety of genes (e.g., actin, globin, and immunoglobulin genes) [57,60–62]. In eukaryotic genomes, potential Z-DNA-forming sequences are abundant, estimated to occur approximately once in every 3,000 base pairs in human genomes [9,63].

Importantly, Z-DNA-forming sequences have been found to be substantially enriched at or near translocation breakpoint hotspots in human cancer genomes, implicating them in cancer etiology [14]. For example, Z-DNA sequences have been found to be located near translocation breakpoints in the *c-MYC* and *BCL-2* genes, implicating them in the development of lymphomas and leukemias [9,64–66]. Z-DNA has also been shown to increase mutations in yeast, bacterial, and mammalian systems [67,68]. In bacteria, Z-DNA was found to increase small deletions within the repeats. In contrast, in mammalian cells, Z-DNA predominantly stimulated the formation of large deletions and rearrangements. Microhomologies were identified at the breakpoint junctions, indicating that a microhomology-mediated end-joining mechanism was involved in the mutagenic processing of Z-DNA-induced DSBs [67]. Consistent with this mechanism, when *E. coli* were engineered to express proteins to reconstitute non-homologous end-joining (which is lacking in wild-type *E. coli* cells), Z-DNA-induced mutations mirrored those found in mammalian cells, with large deletions being ~10-fold more abundant than in wild-type bacterial cells [69]. The mutagenic potential of Z-DNA has also been investigated in vivo via a transgenic mouse model. In this model, mouse oocytes were injected with a linearized reporter-shuttle vector containing either a Z-DNA-forming sequence or a control B-DNA-forming sequence. Strikingly, upon PCR analysis of genomic DNA, 6.6% of the Z-DNA-containing F1 offspring contained large deletions and rearrangements, while none were identified in the control B-DNA mice, demonstrating the mutagenic potential of Z-DNA in vivo [70].

**Figure 5.**
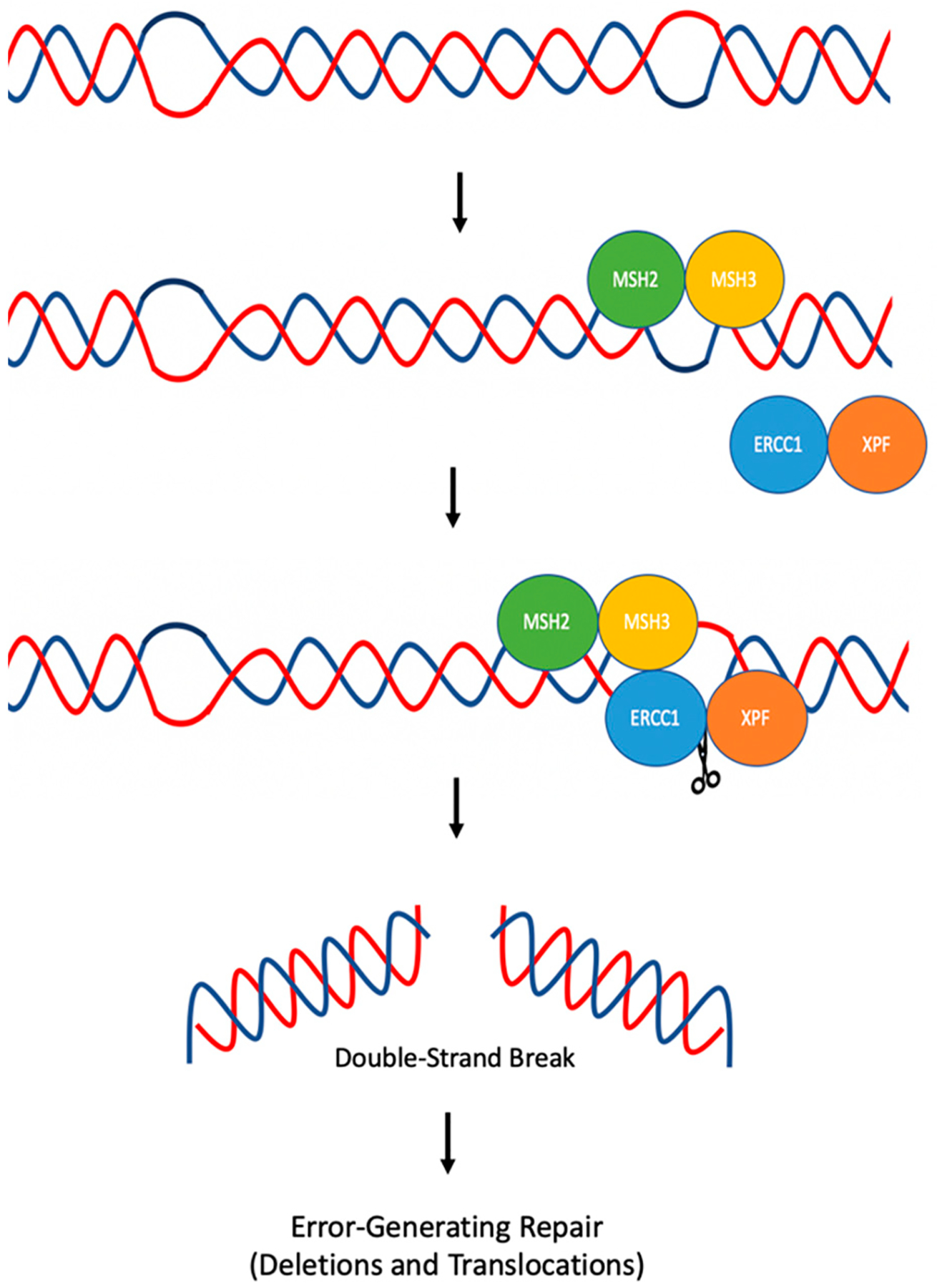
Mechanism of mutagenic Z-DNA processing. MSH2-MSH3 binds to the junction between B-DNA and Z-DNA. ERCC1-XPF is then recruited to the site where it can cleave within and/or surrounding the Z-DNA structure, creating a DSB, which can be repaired in an error-generating fashion, leading to genetic instability. Adapted from McKinney et al. (2020) [68].

Various DNA repair proteins have been identified to be involved in the mutagenic processing of Z-DNA in yeast. Most notable were the NER proteins, Rad1 (XPF) and Rad10 (ERCC1), and the mismatch repair proteins, Msh2 (MSH2) and Msh 3 (MSH3). When examined in human cells using mutation reporters containing a control B-DNA or Z-DNA sequence, the absence of either XPF or MSH2 decreased Z-DNA-induced mutations, implicating these proteins in the mutagenic processing of Z-DNA. This was examined further through chromatin immunoprecipitation (CHIP) assays, where the association of ERCC1-XPF and MSH2-MSH3 were found to be significantly enriched at the Z-DNA region compared to the control B-DNA region. Additionally, results demonstrated that the enrichment of ERCC1-XPF at Z-DNA was dependent on MSH2. A mechanism was proposed, as outlined in Figure 5, in which the MSH2-MSH3 complex recognizes and binds to the Z-DNA-containing region. Upon binding, ERCC1-XPF is then recruited to the Z-DNA structure and cleaves it near the B-Z junction, resulting in a single- or double-strand DNA break. This break can then be repaired in an error-generating fashion, resulting in genetic instability [68].

## 7. H-DNA

H-DNA is a three-stranded DNA structure formed at polypurine (R) and polypyrimidine (Y) DNA sequences with mirror symmetry (Figure 1F). This structure can form during transcription, replication, and repair, when the DNA duplex unravels and a single strand folds back and binds to the major groove of the DNA duplex via Hoogsteen hydrogen bonding. This forms a three-stranded DNA helix as well as a single-stranded DNA section [9,11,71–73]. The third strand of the helix can bind to the purine-rich strand of the underlying DNA duplex to form either Hoogsteen (Y:R:Y) or reverse-Hoogsteen (R:R:Y) hydrogen bonds [11,71,73]. H-DNA-forming sequences are abundant in eukaryotic genomes, with an estimated occurrence of ~1 in every 50,000 base pairs in the human genome [47].

Of clinical relevance, potential non-B DNA structure (PONDS)-forming sequences were analyzed against translocation and deletion breakpoints in human cancer genomes in computational studies, which revealed that H-DNA-forming sequences were significantly enriched at these sites [14,74]. Examples include H-DNA-forming sequences in the *c-MYC* and *BCL-2* oncogenes that co-localize with translocation hotspots in lymphomas and leukemias [9,11,72,74–76]. Wang and Vasquez (2004) investigated the mutagenic potential of H-DNA in COS-7 mammalian cells using a *supF* mutation reporter system. In this study, an H-DNA-forming sequence from the human *c-MYC* gene, which maps to a translocation breakpoint hotspot in Burkitt lymphoma, as well as various other model H-DNA-forming sequences, were used. When transfected into COS-7 cells, the mutation frequencies induced by the H-DNA-forming sequences were up to 20 times higher than those of the control B-DNA sequence, indicating that H-DNA is mutagenic in mammalian cells. When characterized further, it was determined that H-DNA stimulated the formation of DSBs, leading to subsequent deletions [77]. In order to investigate the mutagenic potential of H-DNA in vivo, a transgenic mouse model was constructed in which a recoverable mutation reporter shuttle vector was incorporated into the mouse genome. Through PCR analysis, the mutation frequencies obtained from mice carrying a human H-DNA-forming sequence, from the c-*MYC* promoter region, was significantly higher than mice carrying a control B-DNA sequence. In total, 7.7% of the H-DNA F1 offspring experienced deletions and rearrangements, while none were detected in the B-DNA control mice [70].

**Figure 6.**
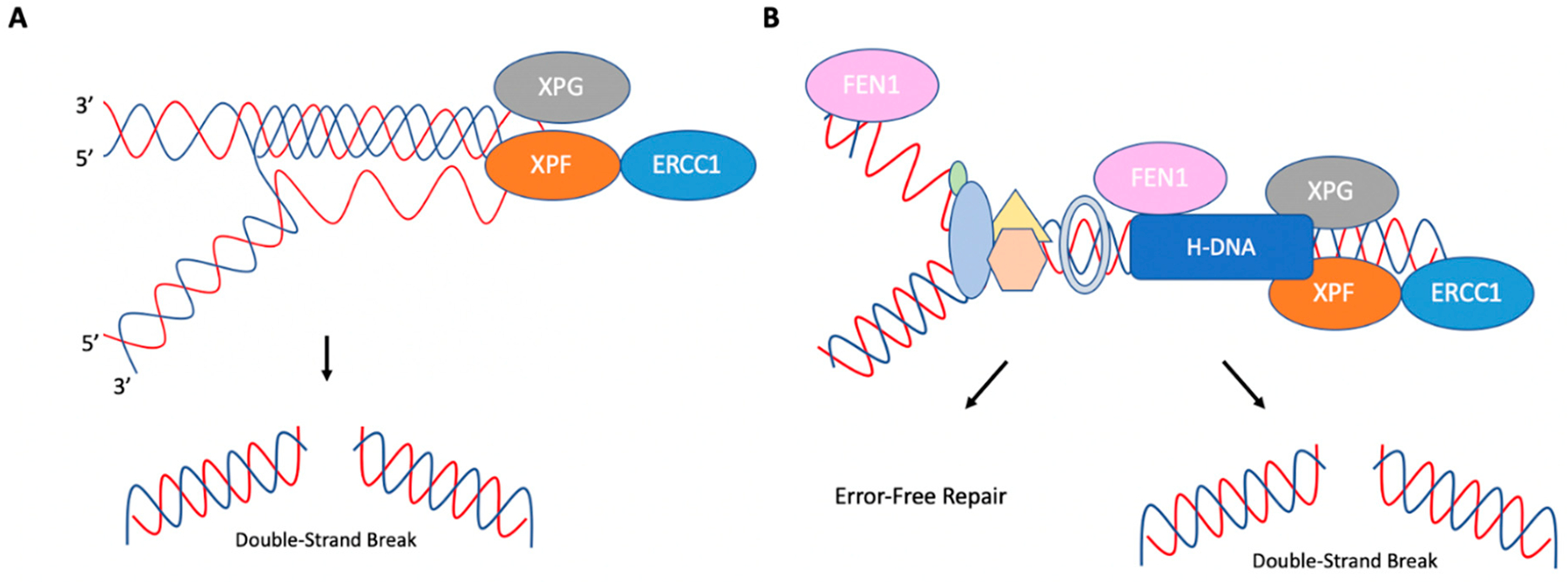
Replication-dependent and replication-independent cleavage of H-DNA. (**A**) In replicationin-dependent processing of H-DNA, ERCC1-XPF and XPG are recruited to H-DNA. ERCC1-XPF cleaves the loop between Hoogsteen hydrogen-bonded strands, and XPG cleaves ssDNA near the DNA duplex. (**B**) In replication-dependent processing of H-DNA, the structure can impede progressing replication complexes. FEN1 can cleave H-DNA to prevent mutations by allowing for continuous replication. However, ERCC1-XPF and XPG may also cleave this structure, which can then be processed in a mutagenic fashion. Adapted from Zhao et al. (2018) [74].

Zhou et al. (2018) found that ERCC1-XPF could cleave H-DNA in both replication-dependent and replication-independent mechanisms (Figure 6) [74]. Rad1 (XPF), Rad10 (ERCC1), and Rad27 (FEN1: flap endonuclease 1) were found to be involved in the processing of H-DNA in yeast and human cells. Interestingly, while depletion of Rad1 (XPF) or Rad10 (ERCC1) decreased H-DNA-induced mutations, a deficiency in Rad27 (FEN1) increased H-DNA-induced mutations. These findings suggest that ERCC1-XPF is involved in the mutagenic processing of H-DNA and FEN1 in its error-free processing. Using a radiolabeled H-DNA-forming substrate in human XPF-proficient and XPF-deficient cell extracts, it was shown that ERCC1-XPF cleaved H-DNA in the loop formed between Hoogsteen hydrogen-bonded strands. Both XPG and FEN1 were found to cleave H-DNA in the single-stranded region near the DNA duplex [74]. Recruitment of Rad27 (FEN1), Rad1 (XPF), and Rad2 (XPG) to H-DNA sequences was confirmed through ChIP assays performed in yeast. Interestingly, depletion of Rad14 (XPA) reduced the recruitment of Rad1 (XPF) to H-DNA, but not Rad27 (FEN1), suggesting that functional NER was involved in the mutagenic processing of H-DNA. This was also supported by mutagenesis assays performed in human XPA-proficient and XPA-deficient cells [74].

The effects of ERCC1-XPF and FEN1 on H-DNA-induced mutagenesis were further investigated in DNA replication-proficient and replication-deficient systems. An increase in H-DNA mutations was observed in both replication-proficient and -deficient systems. However, the absence of XPF impacted H-DNA-induced mutations to a greater extent in non-replicating systems than in replicating systems. In contrast, in replication-proficient systems, FEN1 depletion increased H-DNA-induced mutations, possibly through stabilization of the structure and subsequent processing by ERCC1-XPF and XPG. Thus, two mechanisms were proposed for the processing of H-DNA, as outlined in Figure 6. FEN1 reduces H-DNA-induced genetic instability in a replication-dependent manner by allowing continuous replication through stalled replication forks via cleavage of the H-DNA structure. In contrast, ERCC1-XPF and XPG contributes to the mutagenic processing of H-DNA. In the replication-independent mechanism of H-DNA processing, ERCC1-XPF and XPG are recruited to the structure by XPA and cleave the H-DNA structure, which can lead to subsequent DSBs and genetic instability [74].

## 8. G-Quadruplex (G4 DNA)

Formed in guanine-rich DNA containing a G_≥3_N_x_G_≥3_N_x_G_≥3_N_x_G_≥3_ sequence pattern, G-quadruplexes (G4s) are stacked structures consisting of square planar arrays, with each array consisting of four guanine molecules (Figure 1G). Within each array, a guanine is Hoogsteen hydrogen-bonded to the neighboring guanine [11,78]. G4 DNA can form a variety of intramolecular or intermolecular conformations (e.g., parallel and antiparallel) based on DNA sequence and strand orientation [11,78–81]. Similarly to other non-B DNA structures, cellular functions in which DNA exists in a single-stranded state, such as replication, afford the opportunity for structure formation [78]. As a consequence of structure formation, G4s can also impede replication and transcription complexes, which may increase genetic instability [78,82].

G4-forming sequences have been associated with a variety of human diseases, including ataxia and Fragile X syndrome [83–85]. Nucleotide repeats found in the *FMR1* gene involved in Fragile X syndrome (d(CGG)_n_) have been shown to form G4 DNA, which can alter replication and transcription of the *FMR1* gene [83,84,86]. Additionally, G4-forming sequences have been found to be associated with translocation breakpoints in human cancer genomes [14].

Computational studies have been conducted to determine the prevalence of G4-forming sequences with ~376,000 sequences identified in the human genome [87,88], though more recent studies indicate that there may be as many as 700,000 potential G4-forming sequences [89]. These structures have been found to form in telomeres [78,90–92], immunoglobulin switch regions [11,93], and in promoters [78,94], among other regions. Due to the prevalence of potential G4 DNA-forming sequences in promoter regions of genes [87–89,94], it is thought that G4 DNA plays a role in transcriptional regulation [82,95]. One example is the G4-forming sequence in the promoter region of the *c-MYC* gene [78,96,97]. A G4 DNA-forming sequence in the nuclease hypersensitive element III (NHE III), downstream of the *c-MYC* promoter region, was shown to suppress transcription of *c-MYC* [78,96,97]. It was found that upon disruption of this sequence to prevent G4 structure formation, transcriptional activity increased by several fold [96].

**Figure 7.**
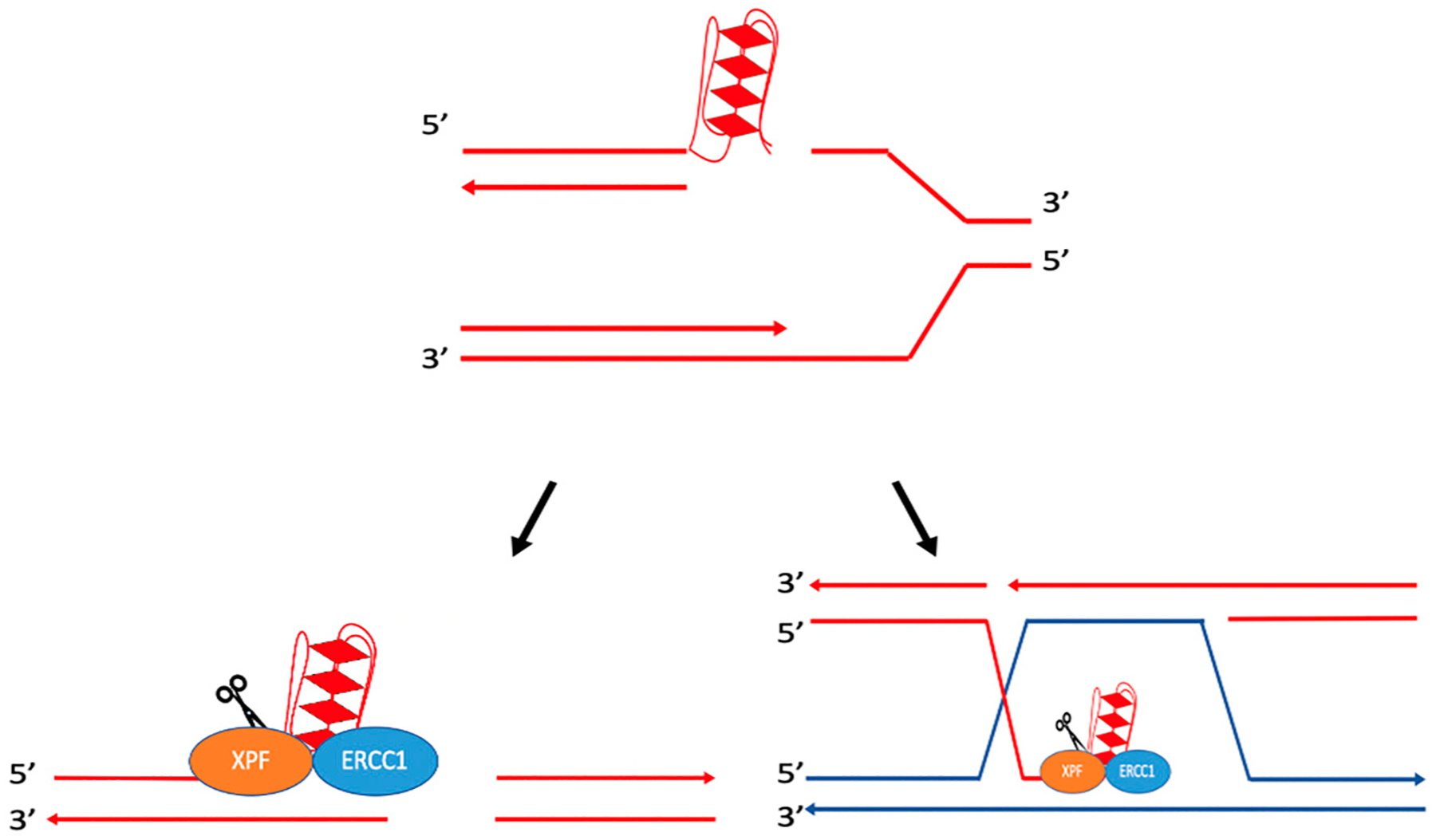
Cleavage of G-quadruplex (G4) DNA by ERCC1-XPF. G4 structure formation may occur during cellular processes, such as replication, causing fork stalling. ERCC1-XPF may cleave G4 structures to allow replication and/or repair to continue. Adapted from Li et al. (2019) [102].

As G4 structures can impede cellular processes such as replication, cells possess mechanisms to resolve these structures. For example, several helicases such as FANCJ [98], BLM [99,100], WRN [99], Sgs1p [100], and Pif1 [101] can resolve these structures. ERCC1-XPF may also process G4 structures formed at DSBs, through cleavage of the structure (Figure 7) [102]. Previously, Wang et al. (2018) found that FANCM prevented DSB formation caused by alternative DNA structures by means of fork reversal [103]. In another study, Li et al. (2019) found that in the absence of FANCM, mitotic recombination induced by alternative DNA structures was dependent on XPF, and absence of both FANCM and XPF resulted in decreased cell proliferation [102]. Using FANCM knockout cells, when treated with pyridostatin (PDS; a small molecule that stabilizes G4 structures), an increase in γH2AX formation was observed, indicating an increase in DSBs. Similar results were obtained in XPF knockout cells. Upon treatment, it was found that XPF knockout cells had a significant increase in DSBs over that of the wild-type cells, suggesting that XPF facilitates the repair of DSBs containing G4 structures [102]. Li et al. (2019) then reduced XPF expression in FANCM knockout cells to a third of its original level, using a lentivirus encoding XPF shRNA, and treated cells with PDS. Inhibition of XPF significantly reduced cell viability in FANCM knockout cells when compared to wild-type cells [102]. Consistent with this study, pyridostatin and TmPyP4, another small molecule known to stabilize G4 structures, were also found to increase DSB formation in neurons [104].

## 9. Concluding Remarks

The repair of DNA damage and/or structural alterations (e.g., non-B DNA) is crucial for maintaining genome integrity. DNA can be damaged by a variety of endogenous and exogenous sources. With the aid of DNA repair proteins, damaged DNA can be repaired in an error-free fashion to reduce genetic instability and prevent the development of diseases such as cancer. One critical protein complex involved in the repair of DNA damage is ERCC1-XPF, an endonuclease that is known for its role in NER and other DNA repair mechanisms. However, ERCC1-XPF has also been found to contribute to the processing of alternative DNA structures in the presence or absence of DNA damage. Here, we reviewed the various roles that ERCC1-XPF plays in the resolution of non-B DNA structures. The studies discussed in this review outline a clear relationship between ERCC1-XPF and non-B DNA structures and provide a mechanistic outline for the processing of these mutagenic DNA structures.
